# Assessment of Fish Protein Hydrolysates in Juvenile Largemouth Bass (*Micropterus salmoides*) Diets: Effect on Growth, Intestinal Antioxidant Status, Immunity, and Microflora

**DOI:** 10.3389/fnut.2022.816341

**Published:** 2022-05-12

**Authors:** Ze Fan, Di Wu, Jinnan Li, Yuanyuan Zhang, Zhiying Cui, Tianbi Li, Xianhu Zheng, Hongbai Liu, Liansheng Wang, Hongqin Li

**Affiliations:** ^1^Key Laboratory of Aquatic Animal Diseases and Immune Technology of Heilongjiang Province, Heilongjiang River Fisheries Research Institute, Chinese Academy of Fishery Sciences, Harbin, China; ^2^Guangdong Xipu Biotechnology Co., Ltd, Guangzhou, China; ^3^Animal Feed Science Research Institute, New Hope Liuhe Co., Ltd, Chengdu, China

**Keywords:** fish protein hydrolysates, largemouth bass, protein synthesis, intestinal immunity, intestinal microflora, intestinal health

## Abstract

Varying dietary inclusion levels of fish protein hydrolysates (FPH) were applied in a feeding experiment with juvenile largemouth bass (*Micropterus salmoides*) to assess their effects on growth, intestinal antioxidant status, immunity, and microflora. FPH were added in 4 dietary levels: 0 g/kg (control group, FPH-0), 10 g/kg (FPH-10), 30 g/kg (FPH-30), and 50 g/kg (FPH-50) dry matter, respectively substituting 0, 5.3, 16.3, and 27.3% of fish meal with dietary fish meal. Quadruplicate groups of 25 juvenile largemouth bass with initial body weight 9.51 ± 0.03 g were fed during the 56-day feeding experiment. Experimental results showed that fish fed FPH-30 obtained a significantly higher weight gain rate (WGR), specific growth rate (SGR), protein efficiency ratio (PER), and significant feed conversion rate (FCR) compared to the other three groups (*P* < 0.05). FPH-30 group also promoted protein synthesis and deposition, as evidenced by the higher whole-body crude protein contents, the higher expressions of GH1, IGF-1, TOR, and S6K in the liver, and SLC7A5, SLC7A8, SLC38A2, and SLC15A2 in the intestine than the other three groups. FPH-30 group could also enhance intestinal health status by increasing the activities of SOD, POD, CAT, GSH-Px, and T-AOC activities by upregulating the expressions of SOD, GSH-Px, IL1β, and TNFβ, and by reducing the MDA contents and the expressions of IL15, Caspase 3, Caspase 9, and Caspase 10 than other groups. Compared to the control group, the *Actinobacteriota* abundance markedly decreased in FPH treatments, while the variation tendency of the phylum *Proteobacteria* was opposite. The peak value of *Firmicutes:Bacteroidetes* ratio and the lowest of *Bacteroidetes* abundance were seen in largemouth bass fed FPH-30 (*P* < 0.05). Fish in three FPH treatments had lower abundances of opportunistic pathogens *Staphylococcus* and *Plesiomonas* than fish in the control group. In conclusion, FPH is a nutritious feed ingredient for juvenile largemouth bass, and can be added to a dietary level of 30 g/kg dry matter replacing fish meal without any negative effect on growth and feed utilization. FPH supplements could also strengthen the intestinal immune mechanisms of largemouth bass to tackle the immunodeficiency produced by fish meal replacement.

## Introduction

Largemouth bass (*Micropterus salmoides*) is a carnivorous fish of considerable high economic value, and has the multiple advantages of strong adaptability, tasty meat, no intramuscular thorn, and abundant nutritional value (1). It has been an important freshwater aquaculture species with total aquaculture production of ~478,000 t in 2019 (2). Numerous studies have revealed that largemouth bass has a higher nutritional requirement for dietary protein. Nowadays, the protein content of the commercial feed of largemouth bass has reached 450 g/kg−516 g/kg, and dosage of fish meal in the commercial feed has reached up to 400–500 g/kg (3, 4), suggesting that largemouth bass has a strong dependence on fish meal. But currently, it has been universally recognized that limited resources and an unstable supply of fish meal cannot continue to meet the needs of the ever-growing aquatic feed industry (5, 6). Hence, it is imminent how to reduce fish meal consumption without suppressing the growth for the healthy and sustainable development of largemouth bass farming industry.

In practice, developing the multifarious alternative protein source is a universal choice for reducing dependence on fish meal (7–10). Currently, partial fish meal replacement by krill meal (11), chicken meal (12), feather meal (13), fermented soybean meal (14), and hydrolyzed yeast protein (15) in largemouth bass feed has been shown to be feasible. However, due to the negative factors such as unbalanced nutrition and antinutritional factors, excessive fishmeal replacement by single plant or animal protein source or novel protein source will result in low-feed utilization rate and poor protein utilization efficiency, thereby affecting the healthy growth of fish (6, 16). In view of the above situation, more and more studies have concentrated on the application prospect of fish processing byproducts in aquatic animal feed (17). Of these, fish protein hydrolysates (FPH), derived from fish and shellfish processing byproducts, have been substantiated to potentially become nutritious ingredients and fish meal substitution in aquatic feed (18, 19). As shared by Valentina et al. (20), the hydrolysate product of anchovies (*Engraulis encrasicolus*) included differently sized peptides and free amino acids, accordingly being endowed with great potential as a promising biological and nutritional substance.

In the current studies, FPH have been testified to be the valuably nutritious ingredients for different sort of marine aquacultured species, including Atlantic salmon (21), red sea bream (22), olive flounder (22), European sea bass (23), Atlantic cod (24), large yellow croaker (25), whereas so far, the application study of FPH for freshwater aquacultured species has been only conducted on rainbow trout (26), African catfish (27), and yellow catfish (28). Moreover, there has been still scarcely any information available to assess the application effect of FPH hydrolyzed protein in largemouth bass feed. Existing studies have revealed that appropriate fish protein hydrolysate supplementation in aquatic feeds effectually contributes to the growth performance, efficiency of feed utilization, and regulation of the lipid accumulation and fatty acid compositions (29). Besides these, FPH also has immune-boosting effects, which are involved in improving the oxidative function and disease resistance of fishes. This has been found in the study of red seabream (30), which indicated that the increased disease resistance by the hydrolysates supplementation can attribute to the significant enhancement of immune responses of the liver, including superoxide dismutase (SOD), lysozyme and anti-protease activities, and total Ig level, which is in line with these results in a research on African catfish (27). In spite of the enhancement of immune responses of the liver, scarce knowledge is getable concerning the effect of FPH on fish intestinal health, which plays a crucial role in nutrient absorption and host-defense to hostile environment and poor nutritional conditions for fish (31).

Considering the above research status, this study was designed to assess the effects of FPH supplementation for largemouth bass (*Micropterus salmoides*) on growth performance, intestinal peptide and amino acid transporters, intestinal antioxidant status, immunity, and microflora in a 56-day experiment. We believe the outcomes of this study will make a significative contribution to the needs of the increasing largemouth feed industry and thereby fulfill the sustainable development of largemouth bass aquaculture.

## Materials and Methods

### Preparation and Nutritional Composition of Experimental Diets

Fish protein hydrolysates (FPHs) was purchased from Guangdong Xipu Biological Technology Co., LTD. The molecular weights of the hydrolysates were detected by high performance liquid chromatography (HPLC) with a TSK G2000 column, according to GB/T22492-2008 (32). Polypeptides with molecular weight <1,000 Da accounted for 81.1%, 1,000 -−2,000 Da accounted for 13.4%, and 2,000–5,000 Da accounted for 5.5% in FPH powder.

The ingredients and biochemical composition of the experimental diets, based on the nutritional requirement of largemouth bass (1) are listed in Table 1. The amino acid composition of experimental diets is presented in Table 2. Four isonitrogenous (500 g/kg crude protein) and isolipidic (120 g/kg crude lipid) experimental diets were designed to include fish meal, chicken powder, soybean meal, soybean protein concentrate, and cottonseed protein as dietary protein source as well as fish oil and soybean oil as dietary lipid source, which differed in the supplemental level of fish meal and FPHs (Table 1). The experimental diets were produced containing the step-up levels of FPHs (0, 10, 30, and 50 g/kg), which correspondingly substituted 0, 5.3, 16.3, and 27.3% of fish meal of total dietary protein. All dry feed ingredients were kneaded into a smooth dough by adding fish oil, soybean oil, and water. The dough was produced into pellets by a pelletizer (GYJ-250B, Dashiqiao Bao's Feed Machinery Factory) and dried for 12 h in a ventilated oven at 55°C. Then the dry diets were smashed into proper pellet size (3.0 × 3.0 mm), and were kept at −20°C before use.

**Table 1:** Ingredients and proximate composition of the experimental diets (g/kg of dry matter basis).

**Ingredients**	**Diets**			
	**FPH-0**	**FPH-10**	**FPH-30**	**FPH-50**
Fish protein hydrolysates^a^	0	10	30	50
Fish meal^b^	300	284	251	218
Chicken powder^b^	150	150	150	150
Soybean meal^b^	58	58	58	58
Soybean protein concentrate^b^	160	160	160	160
Conttonseed protein^b^	100	100	100	100
Wheat middling^a^	115	121	134	147
Fishoil	50	50	50	50
Soybean oil	35	35	35	35
Vitamin premix^c^	5	5	5	5
Trace mineral premix^d^	3	3	3	3
Choline chloride	20	20	20	20
Dicalcium phosphate	4	4	4	4
Crude protein	501.8	501.8	501.4	500.0
Crude lipid	122.9	123.2	121.8	121.4
Crude Ash	66.0	65.5	64.4	64.3

a *Fish protein hydrolysates: crude protein 908.6 g/kg dry matter, crude lipid 2.0 g/kg dry matter*.

b *Fish meal: crude protein 670.0 g/kg dry matter, crude lipid 48.5 g/kg dry matter; Chicken powder: crude protein 660.0 g/kg dry matter, crude lipid 130.0 g/kg dry matter; Soybean meal: crude protein 440.0 g/kg dry matter, crude lipid 15.0 g/kg dry matter; Soybean protein concentrate: crude protein 630.0 g/kg dry matter, crude lipid 5.0 g/kg dry matter; Conttonseed protein: crude protein 635.0 g/kg dry matter, crude lipid 18.0 g/kg dry matter; Wheat middling: crude protein 130.0 g/kg dry matter, crude lipid 12.0 g/kg dry matter*.

c *Vitamin mixture (g/kg mixture) supplied by Guangdong Hyint Biotechnology Group Co. Ltd: vitamin A(VA) 8,000 IU, vitamin C (VC) 100 mg, vitamin D_3_ (VD_3_) 3,000 IU, vitamin E (VE) 60 mg, vitamin K_3_ (VK_3_) 5 mg, vitamin B_1_ (VB_1_) 15 mg, vitamin B_2_ (VB_2_) 30 mg, vitamin B_6_ (VB_6_) 15 mg, vitamin B_12_ (VB_12_) 0.5 mg*.

d *Trace mineral mixture (mg/g mixture) supplied by Guangdong Hyint Biotechnology Group Co. Ltd: nicotinamide 175 mg, d-biotin 2.5 mg, inositol 1,000 mg, folic acid 5 mg, pantothenic acid 50 mg, zinc (Zn) 60 mg, copper (Cu) 3 mg, iron (Fe) 25 mg, manganese (Mn) 15 mg, iodine (I) 0.6 mg, and magnesium (Mg) 0.7 mg*.

**Table 2:** Amino acid composition of experimental diets (g kg^−1^ dry diet).

	**FPH-0**	**FPH-10**	**FPH-30**	**FPH-50**
**Essential amino acid**				
Methionine	13.06	13.01	12.90	12.80
Lysine	29.43	29.31	29.05	28.78
Arginine	36.96	36.85	36.59	36.33
Histidine	11.43	11.37	11.23	11.09
Isoleucine	22.66	22.48	22.09	21.71
Leucine	40.84	40.54	39.90	39.26
Phenylalanine	24.74	24.58	24.25	23.91
Threonine	20.28	20.18	19.94	19.70
Valine	27.48	27.31	26.94	26.57
**Non-essential amino acid**				
Alanine	29.11	29.01	28.78	28.54
Glutamic acid	89.05	88.47	87.20	85.93
Glycine	35.39	35.68	36.20	36.72
Aspartic acid	44.41	44.49	44.59	44.70
Serine	24.75	24.79	24.86	24.92
Tyrosine	15.31	15.17	14.86	14.56
Proline	35.42	35.37	35.23	35.08

### Experimental Fish and Husbandry

Acquired from Hulan Experimental Station of Heilongjiang Fisheries Research Institute (Harbin, China), all the juvenile largemouth bass was fed with commercial feed (50 g/kg dry matter, crude lipid 120 g/kg dry matter) twice per day for 2 weeks to acclimate to the experimental conditions before the experiment. After acclimation, fish of similar sizes (9.51 ± 0.03 g) were randomly allotted into 16 indoor aquarium (a water volume of 200 L) with a recirculating aquaculture system and an auto-supplemented oxygen system, and each aquarium was stocked with 25 fish. Each diet was randomly designated to quadruplicate aquarium, and concurrently hand-fed to all the largemouth bass at the rate of 7–8% of the body weight, three times a day (8:00 a.m., 13:00, and 17:30). Throughout the 8-week husbandry trial, the multiparameter water quality analyzer (HQ40D) (HACH Company, America) was applied to monitor the main water quality parameters, including the water temperature (28–29°C), total ammonia (0–0.20 mg/L), and dissolved oxygen (5.8–6.2 mg/L).

### Calculations of Growth Indices

At the end of the 8-week husbandry trial, all fish from each aquarium were weighed and counted to calculate the main growth indices after 24 h fasting according to the following computational formula.

Survival rate (SR, %) = 100 × final number (fish)/initial number (fish)

Weight gain rate (WGR, %) = 100 × [final weight(g)-initial weight (g)]/final weight (g)

Specific growth rate (SGR, %/d) = 100 × [(ln final weight- ln initial weight)/56 days]

Feed intake (FI, g/fish) = total feed intake per aquarium (g)/(number of fish^*^56 days)

Feed conversion rate (FCR) = 100 × dry feed intake (g) / body weight gain (g)

Protein efficiency ratio (PER, %) = 100% × [(final weight (g)-initial weight (g)) /(total feed intake (g) × content of dietary protein (%)]

### Sample Collection and Assay Method for Proximate Composition of Fish and Diets and Enzymatic Activities in the Intestine and Stomach

Six fish from each aquarium (24 fish per diet treatment) were randomly chosen and ground for next proximate composition analysis of the whole body. Other three fish per aquarium (24 fish per diet treatment) were anesthetized with MS-222 (0.1 g/L) (33) and dissected to acquire the intestine and stomach for assessing the related enzyme activity. The samples were ground with a homogenizer in nine volumes of ice-cold physiological saline (0.9% w/v NaCl) and centrifuged with 3,500 rpm for 15 min at 4°C (Thermo Fisher Scientific ST 16R centrifuge). Then the resulting supernatants were aliquoted and kept at −20°C until further analysis. The above detection details are presented in Table 3.

**Table 3:** The chemical analysis used in the experiment.

**Items**	**Methods (NO.)**	**Reference/Assay kits**
**Composition of diets**		
Moisture	Drying at 105°C to constant weight	(34)
Crude protein	Kjeldahl method	
Crude lipid	Ether extraction method with a Soxtec system	
Crude ash	Combustion to a constant weight at 550°C	
**Activities of digestive enzymes in the intestine and stomach**		
Trypsin	Ultraviolet colorimetry (A080-2-2)	Assay kits purchased from Jian Cheng Bioengineering Institute (Nanjing, China)
Lipase	Ultraviolet colorimetry (A054-1-1)	
Amylase	Ultraviolet colorimetry (C016-1-1)	
Alkaline phosphatase (AKP)	Microplate method (A059-2-2)	
Acid phosphatase (ACP)	Microplate method (A060-2-2)	
**Intestinal activities of antioxidant enzymes**		
Superoxide dismutase (SOD)	WST-1 method (A001-3-2)	
Catalase (CAT)	Ammonium molybdate method	
Peroxidase (POD)	Ultraviolet colorimetry (A084–1-1)	
Glutathione peroxidase (GSH-Px)	Ultraviolet colorimetry (A005-1-2)	
Total antioxidant capacity (T-AOC)	ABTS method (A015-2-1)	
Malondialdehyde (MDA)	Thiobarbituric acid (TBA) method (A003-1-2)	

### Sample Collection and Assay Method for Gene Expression and Intestinal Microflora Analysis

Another three fish were sampled randomly from each aquarium for obtaining three liver and intestine samples. Immediately, all the above samples were transferred into liquid nitrogen and then stored at −80°C for further analysis of gene expression. Liver and intestine samples were pulverized with liquid nitrogen, and then conducted to extract total RNA with RNAiso Plus kit (TaKaRa Biotechnology Co., Ltd., Dalian, China) according to the manufacturer's protocol. The integrity of RNA samples was verified by 1% agarose gel electrophoresis based upon 28S:18S ratio, whereas the purity of RNA samples based upon the A260/A280 ratio (1.8-2.0) and the concentration of RNA samples were detected using a NanoDrop 2000 (Thermo, USA). To synthesize cDNA template, the obtained total RNA samples were diluted to 1,000 ng/μl, and then heated at 42°C for 2 min to remove genomic DNA, at 37°C for 15 min to generate cDNA, and finally at 85°C for 5 s to inactivate reverse transcriptase following the specification of TaKaRa PrimeScript^TM^ RT reagent Kit with gDNA Eraser (Perfect Real Time) (Code No: RR047A) (TaKaRa Biotechnology Co., Ltd., Dalian, China) and PrimeScriptTM RT reagent Kit with gDNA Eraser (Perfect Real Time) (Code No: RR047A) (Dalian Takara Company). Targeted gene expression levels in the cDNA templates were detected in a 20-μl volume containing 10 μl SYBR^®^ Premix DimerEraser (2 × ), 2 μl cDNA template (≈100 ng), 0.4 μl PCR forward primer, 0.4 μl PCR reverse primer, 7 μl RNase free dH_2_O following the manufacturer's protocol of the TaKaRa SYBR^®^ Primix Ex TaqTM (Tli RNaseH Plus) (Code No: RR420A) on ABI 7500 real-time PCR machine (ABI, Applied Biosystems, USA). Primer sequences for genes used in this study were synthesized according to the previous studies on largemouth bass (35–38) and the existing sequence in NCBI (serial number for SLC15A1 (PepT1): XM_038720562.1) (Table 4). Based on the specific gene standard curves, the amplification efficiency of the target and the housekeeping gene (β-actin) was calculated, which were generated from 10-fold serial dilutions. The thermal cycle program of quantitative real-time PCR was as follows: 95°C for 30 s (predegeneration stage), 40 cycles of a two-step amplification program (95°C for 5 s, 60°C for 34 s) (cycling stage), and then 95°C for 15 s, 60°C for 1 min, 95°C for 30 s, and 60°C for 15 s (Melt curve stage). The comparative CT method (2^−Δ*ΔCt*^) method was used to calculate the relative expression levels of each gene (39).

**Table 4:** Primers used for the quantitative RT-PCR (qPCR).

**Gene name**	**Primer sequence (5^**′**^-3^**′**^)**
**Relative gene expressions of the GH/IGF-1 axis and TOR signal pathway in the liver**
GH^a^	F:CCCCCAAACTGTCAGAACT R:ACATTTCGCTACCGTCAGG
IGF-1^b^	F: CTTCAAGAGTGCGATGTGC R:GCCATAGCCTGTTGGTTTACTG
TOR^c^	F:TCAGGACCTCTTCTCATTGGC R:CCTCTCCCACCATGTTTCTCT
PI3K^d^	F:AAGACCTTCCTCATCACGAC R:CCTTCCACTACAACACTGCA
S6K^e^	F:GCCAATCTCAGCGTTCTCAAC R:CTGCCTAACATCATCCTCCTT
**Immune Response Related Gene Expression in Intestine**	
SOD^f^	F:TGGCAAGAACAAGAACCACA R:CCTCTGATTTCTCCTGTCACC
GSHPx^g^	F:GGGGCTCCACCTGCTTCTTG R:ACCCCTCTGCTCAGGCATTT
IL1β^h^	F:CGTGACTGACAGCAAAAAGAGG R:GATGCCCAGAGCCACAGTTC
IL15^i^	F:GTATGCTGCTTCTGTGCCTGG R:AGCGTCAGATTTCTCAATGGTGT
TNFβ^j^	F:GCTCAAAGAGAGCGAGGATG R:TCCTCTACCATTCGCAATCC
Caspase 3	F:GCTTCATTCGTCTGTGTTC R:CGAAAAAGTGATGTGAGGTA
Caspase 9	F:CTGGAATGCCTTCAGGAGACGGG R:GGGAGGGGCAAGACAACAGGGTG
Caspase 10	F:CAAACCACTCACAGCGTCTACAT R:TGGTTGGTTGAGGACAGAGAGGG
**Relative peptide and AA transporters**
SLC7A5^k^	F:CCAAAGCACGACAGACCTACA R:ACCAACCTGGCATATTTCACC
SLC7A8^l^	F:GGTGACCACAGGGATAGAGATG R:TTGCTTACGGAGGCTGGAACTT
SLC38A2^m^	F:AATAGGGAAAAGCACCACGGG R:GTATGAGGAGCTCAAAGACCG
SLC15A1 (PepT1)^n^	F:AAAGCAGGCAGCACCTTCACTC R:CCTCTCGCAGAACTCATTCACAAC
SLC15A2^o^	F:TGCACATCCCCTCTCAGTACG R:CAAGTCAGTTGGAGCCATTCC
β-actin	F:ATCGCCGCACTGGTTGTTGAC R:CCTGTTGGCTTTGGGGTTC

*^a^GH: Growth hormone; ^b^IGF-1: Insulin growth factor 1 (IGF-1); ^c^TOR: target of rapamycin; ^d^PI3K: phosphatidylinositol 3-hydroxy kinase; ^e^S6K: S6 Kinase; ^f^SOD: superoxide dismutase; ^g^glutathion peroxidase; ^h^IL1β: interleukin 1β; ^i^IL15: interleukin 15; ^j^TNF β: tumor necrosis factor β; ^k^SLC7A5: solute carrier family 7, member 5; ^l^SLC7A8: solute carrier family 7, member 8; ^m^SLC38A2: solute carrier family 38, member 2; ^n^SLC15A1 (PepT1): solute carrier family 15, member 1, oligopeptide transporter 1, PepT1; ^o^SLC15A2: solute carrier family 15, member 2, oligopeptide transporter 2, PepT2*.

Meanwhile, the intestinal contents of the above three fish from the same aquarium were collected, mixed into one, frozen with liquid nitrogen, and stored at −80°C for later analyses of intestinal microflora communities after 7 h of feeding when their feed digestion was almost accomplished and the feces had reached the hindgut. the intestinal contents samples were transported to Shanghai Majorbio Bio-pharm Technology Co., Ltd., immediately under a low-temperature environment of solid carbon dioxide (dry ice). DNA amplification and sequencing were conducted according to the manufacturer's protocol for the extraction of microbial DNA from intestinal contents samples using the E.Z.N.A stool DNA Kit (Omega Bio-Tek). Following the designated measurement area, the conserved sequence of the 16S rDNA gene was designed, where the barcode is an eight-base sequence unique to each sample. The qPCR reaction conditions are as follows: 95°C for 2 min, followed by 30 cycles at 95°C for 20 s, 62°C for 20 s and 72°C for 30 s and a final extension at 72°C for 3 min. PCR reactions were performed in triplicate 50 μl mixtures containing 10 μl of 5 × TransStart^®^ FastPfu buffer, 5 μl of 2.5 mM dNTPs, 2 μl of each primer (10 μM), 1 μl of TransStart^®^ FastPfu DNA polymerase, and 100 ng of template DNA. The product was detected by 2% agarose gel electrophoresis and purified using the AxyPrep DNA Gel Extraction Kit (Axygen Biosciences) according to the manufacturer's instructions. The purified sample was obtained using the QuantiFluor-ST (Promega, US). Purified amplicons were pooled in equimolar and paired-end sequences (2 × 250) on an Illumina platform according to standard protocol. Illumina paired-end reads were joined, filtered, and then performed using the software package of QuantiFluor™-ST.

### Statistical Analysis

The results were presented as the means ± standard deviation (SD*)*. All data were subjected to a one-way analysis of variance (ANOVA) by Duncan's multiple range tests to evaluate significant differences among the treatments at *p* < 0.05 using SPSS statistics version 22.0 (SPSS Inc., Chicago, IL, USA.

## Results

### Growth Performance and Feed Utilization

The survival rates of experimental fish were between 98.67 and 100.00%, and no significant difference was observed among all treatments (*P* > 0.05). Final body weight (FBW), WGR, SGR, and PER in fish fed the FPH-30 diet were significantly higher than those fish fed FPH-0, FPH-10, and FPH-50 diets (*p* < 0.05), while FCR in fish fed the FPH-30 diet were significantly lower than that of fish fed the FPH-0, FPH-10, and FPH-50 diets (*p* < 0.05), although no differences in those growth and feed utilization results were observed for fish fed the FPH-0, FPH-10, and FPH-50 diets (*p* > 0.05). Feed intake was not significantly affected by increasing FPH levels (*p* > 0.05) (Table 5).

**Table 5:** Growth performance and feed utilization of largemouth bass (*Micropterus salmoides*) fed the diets containing different supplementation levels of fish protein hydrolysate (FPH)^*^.

**Items**	**Diets**			
	**FPH-0**	**FPH-10**	**FPH-30**	**FPH-50**
IBW^1^	9.49 ± 0.03	9.50 ± 0.01	9.53 ± 0.01	9.51 ± 0.01
FBW^2^	59.07 ± 1.10^b^	57.69 ± 1.34^b^	68.49 ± 2.28^a^	58.07 ± 0.70^b^
SR^3^	100.00 ± 0.00	98.67 ± 1.33	100.00 ± 0.00	98.67 ± 1.33
WGR^4^	522.61 ± 24.38^b^	522.66 ± 4.82^b^	599.23 ± 25.71^a^	502.35 ± 4.75^b^
SGR^5^	3.26 ± 0.07^b^	3.27 ± 0.01^b^	3.47 ± 0.07^a^	3.20 ± 0.01^b^
PER^6^	2.22 ± 0.08^b^	2.19 ± 0.03^b^	2.53 ± 0.12^a^	2.20 ± 0.05^b^
FI^7^	0.78 ± 0.01	0.80 ± 0.01	0.80 ± 0.01	0.77 ± 0.03
FCR^8^	0.89 ± 0.03^a^	0.90 ± 0.01^a^	0.79 ± 0.04^b^	0.90 ± 0.02^a^

* *Values are the mean ± SD (n = 3) of three replicates. Values within the same row with different superscript small letters are significantly different (P < 0.05).*

*^1^Initial mean body weight (IBW, g per fish); ^2^Final mean body weight (FBW, g per fish); ^3^Survival rate (SR, %); ^4^Weight gain rate (WGR, %); ^5^Protein efficiency ratio (PER, %); ^6^Specific growth rate(SGR, %/d); ^7^Feed intake (FI, g/fish); ^8^Feed conversion rate (FCR)*.

### Proximate Composition of the Whole-Body

The effects of dietary FPH levels on the proximate composition of largemouth bass are listed in Table 6. The crude protein content of the whole-body of fish fed FPH-30 diets was significantly higher than the FPH-50 group (*p* < 0.05). Fish fed FPH-10 had the lowest crude lipid content among the 4 treatments, albeit with no significant difference (*p* > 0.05). There were no significant differences in moisture and ash in all groups (*P* > 0.05).

**Table 6:** The whole-body compositions of largemouth bass (*Micropterus salmoide*s) fed the diets containing different supplementation levels of fish protein hydrolysate (FPH)^*^.

**Items**	**Diets**			
	**FPH-0**	**FPH-10**	**FPH-30**	**FPH-50**
Crude protein/%	18.83 ± 0.02^ab^	18.90 ± 0.02^ab^	19.47 ± 0.40^a^	18.49 ± 0.17^b^
Crude lipid/%	9.67 ± 0.29	9.75 ± 0.20	8.94 ± 0.32	9.26 ± 0.30
Moisture/%	68.12 ± 0.25	67.94 ± 0.60	68.79 ± 0.33	74.53 ± 1.74
Ash/%	3.91 ± 0.04	3.88 ± 0.01	3.86 ± 0.09	3.87 ± 0.09

* *Values are the mean ± SD (n = 4) of three replicates. Values within the same row with different superscript small letters are significantly different (P < 0.05)*.

### Antioxidant Capacity in the Intestine

The antioxidant capacity in the intestine of largemouth bass fed different diets is shown in Table 7. Overall, FPH-30 group enhanced antioxidant enzyme activities and inhibited the accumulation of MDA. Compared with the control group, fish fed diets with 30 g/kg FPH had a significantly higher SOD activity and T-AOC contents and lower MDA contents in the intestine (*P* < 0.05) compared with the FPH-50 group. Additionally, in the intestine of largemouth bass, a significant increment in antioxidant enzyme activities and a significant decrease in MDA contents were observed with the fish in the FPH-50 group (*p* < 0.05).

**Table 7:** Antioxidant capacity in the intestine of largemouth bass (*Micropterus salmoide*s) fed the diets containing different supplementation levels of fish protein hydrolysate (FPH)^*^.

**Items**	**Diets**			
	**FPH-0**	**FPH-10**	**FPH-30**	**FPH-50**
SOD (U/mgprot)	7.15 ± 0.10^b^	8.05 ± 0.60^ab^	8.43 ± 0.23^a^	8.25 ± 0.10^b^
CAT (U/mgprot)	2.28 ± 0.03^ab^	2.34 ± 0.17^ab^	2.39 ± 0.14^a^	1.94 ± 0.12^b^
POD (U/mgprot)	3.92 ± 0.17^a^	3.19 ± 0.10^b^	4.09 ± 0.15^a^	2.53 ± 0.12^c^
GSH (μmol/gprot)	20.65 ± 2.14^ab^	24.06 ± 2.02^ab^	27.12 ± 2.22^a^	17.40 ± 3.20^b^
AOC (mmol/L)	0.18 ± 0.02^b^	0.22 ± 0.05^ab^	0.31 ± 0.03^a^	0.19 ± 0.04^b^
MDA (nmol/mgprot)	2.99 ± 0.21^a^	1.70 ± 0.20^b^	1.53 ± 0.23^b^	2.06 ± 0.12^b^

* *Values are the mean ± SD (n = 4) of three replicates. Values within the same row with different superscript small letters are significantly different (P <0.05)*.

### Relative Gene Expressions of the GH/IGF-1 Axis and TOR Signal Pathway in the Liver

**Figure 1:**
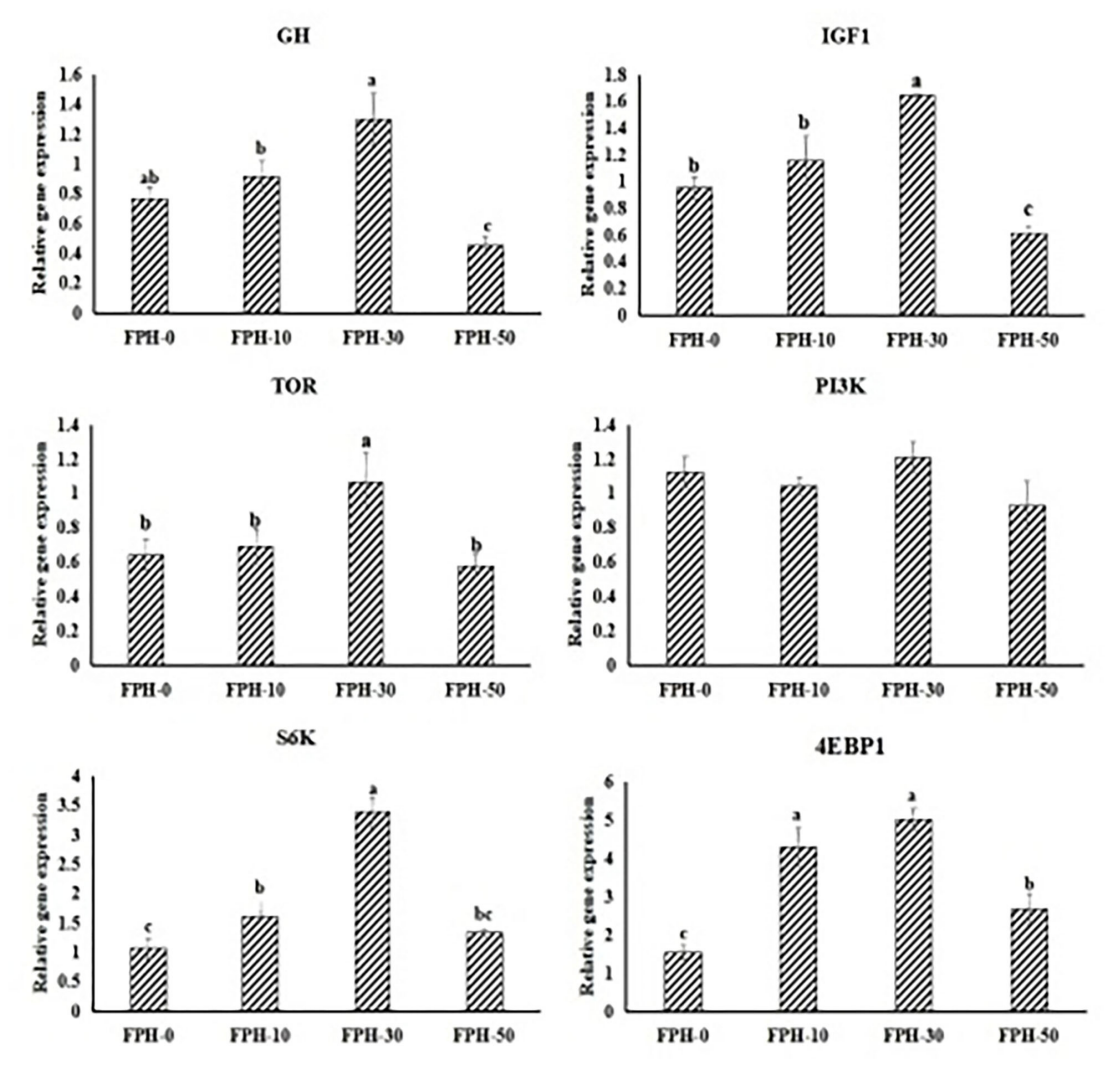
Relative gene expressions of the GH/IGF-1 axis and TOR signal pathway in the hepatopancreas of largemouth bass (*Micropterus salmoides*) fed the diets containing different supplementation levels of fish protein hydrolysate (FPH). Lowercase letters (a, b, c or d) indicate a significant effect of relative gene expressions of the GH/IGF-1 axis and TOR signal pathway in hepatopancreas (*P* < 0.05), and the other figures are the same as in this figure.

Regarding the expression of GH/IGF-1 axis related genes in the liver, relative expressions of GH in fish fed the FPH30 diet was significantly higher than that of fish fed the FPH-10 and FPH-50 diets (*P* < 0.05), but was not significantly different compared with fish fed the FPH-0 diets (*P* > 0.05). Fish fed the FPH30 diet exhibited higher relative expressions of IGF1 in fish compared with fish fed the other three diets (*P* < 0.05). For TOR signal pathway in the liver, the expression of TOR and S6K in fish fed the FPH-30 diet was significantly higher than those of fish fed the other three diets (*p* < 0.05). Fish fed the FPH-30 diet obtained a significantly higher mRNA level of 4E-BP1 in the liver compared with fish fed the FPH-0 and FPH-50 diet (*p* < 0.05), although no significant difference was observed between the FPH-10 and FPH-30 groups (*P* > 0.05). However, no significant difference was found in PI3K mRNA level among all the treatments (*p* > 0.05) (Figure 1).

### Immune-Response Related Gene Expression in the Intestine

**Figure 2:**
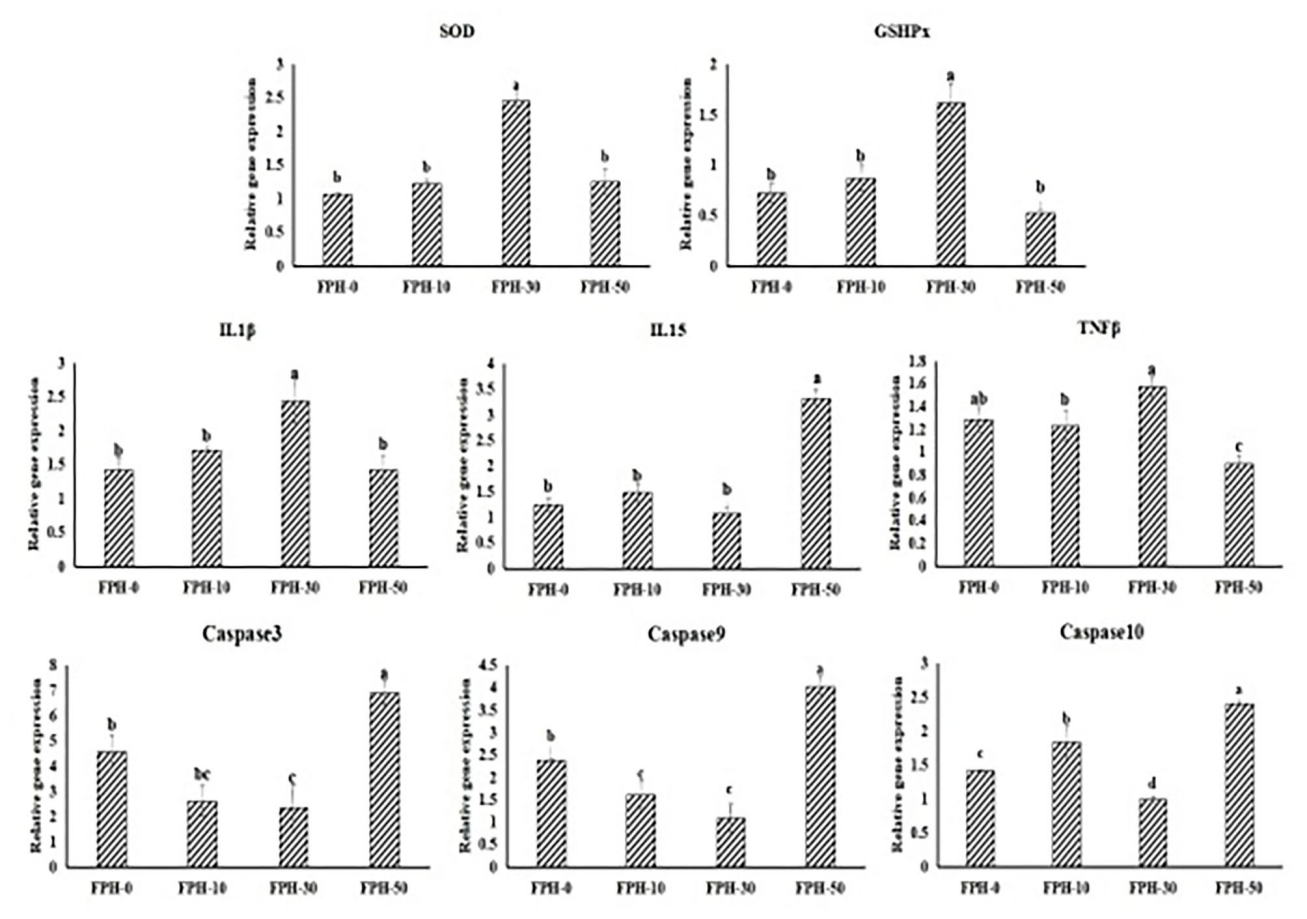
Relative expression of immune response related gene in the intestine of largemouth bass (*Micropterus salmoides*) fed the diets containing different supplementation levels of fish protein hydrolysate (FPH).

Figure 2 shows the relative expression of immune response-related gene expression in the intestine. To antioxidant genes and cytokines genes, SOD, GSHPx, IL15 relative expressions in the FPH30 group were significantly lower compared with the other three groups (*p* < 0.05). Although IL-1β and TNFβ relative expressions in the FPH-30 group are significantly higher compared to the FPH-10 and FPH-50 groups (*p* < 0.05), no significant difference was observed between the FPH-0 and FPH-30 groups (P>0.05). Also, in the caspase family, the lowest values of the relative gene expression of Capase3, capase9, and Capase10 were observed in the FPH-30 treatment (*P* < 0.05). Meanwhile, it was noted that the lowest values of the relative gene expression of Capase3, Capase9, and Capase10 were observed in the FPH-0 treatment, although there was no significant difference with the FPH-50 treatment (*p* > 0.05).

### Gene Expression to Relative Peptide and AA Transporters of Amino Acids Response (AAR) Signaling

**Figure 3:**
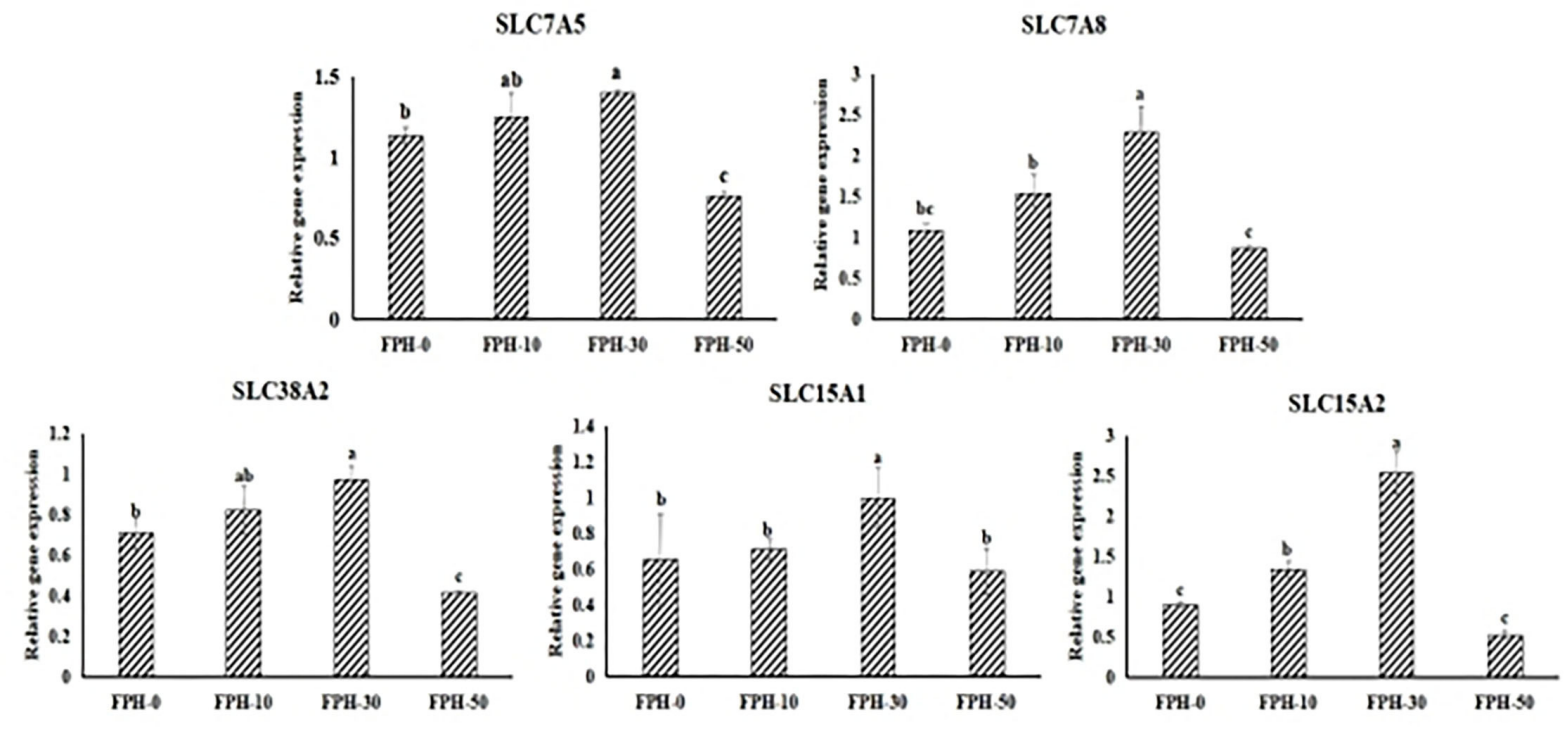
Relative expression of relative peptide and AA transporters related genes in the intestine of largemouth bass (*Micropterus salmoides*) fed the diets containing different supplementation levels of fish protein hydrolysate (FPH).

The gene expression levels of peptide and AA transporters are presented in Figure 3. There were significant increasing relative expression levels of SLC7A5, SLC7A8, SLC38A2, and SLC15A2 in the intestine of fish fed the FPH-30 diet than the fish fed with control diet (*p* < 0.05). Meanwhile, it was noted that the lowest values of the relative gene expression of SLC7A5, SLC7A8, SLC38A2, and SLC15A2 were observed in the FPH-50 treatment (*p* < 0.05).

### Intestinal Microflora Community in the Intestine

#### Diversity Analysis of Intestinal Microflora

**Figure 4:**
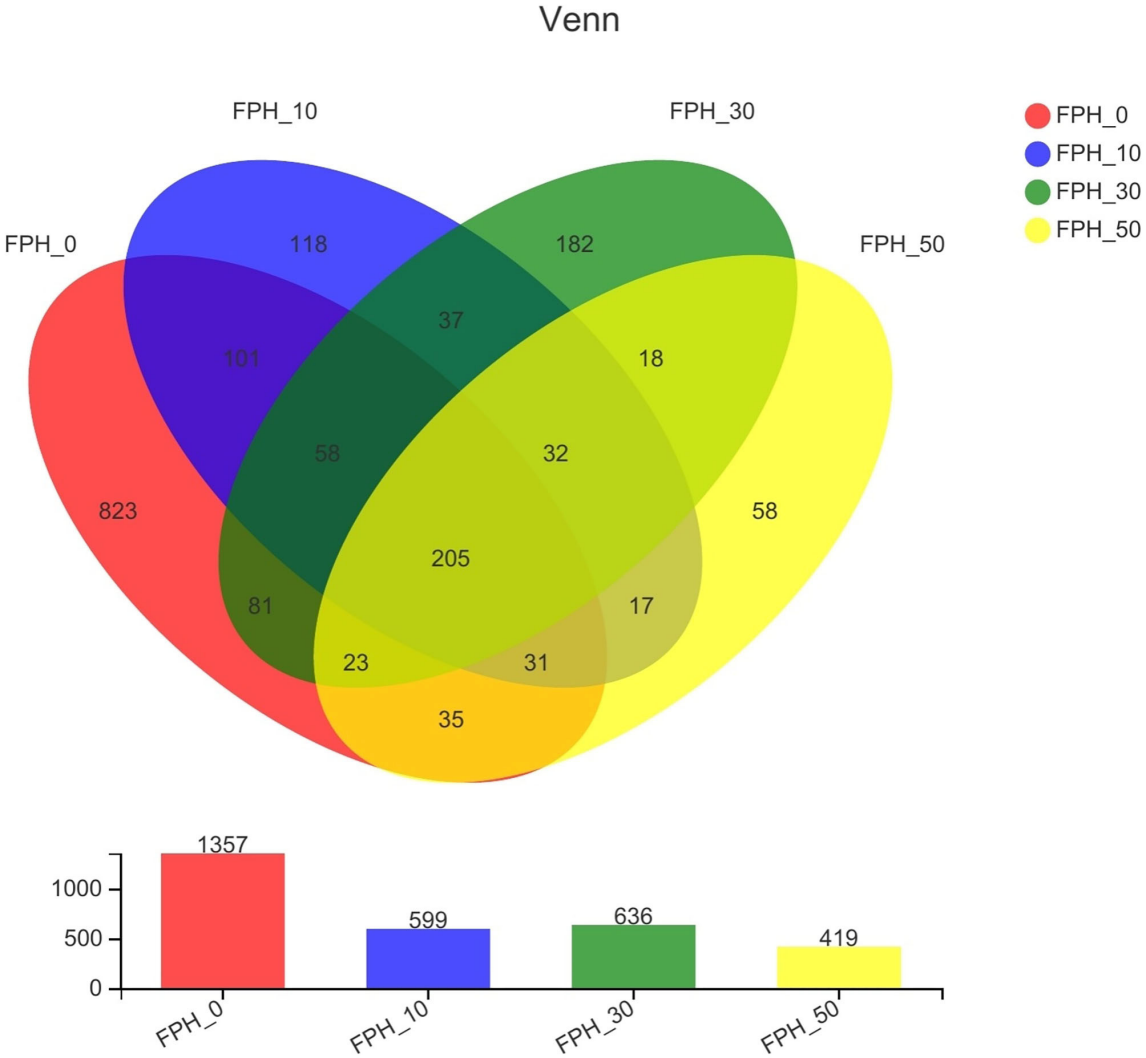
The Venn diagram displays the overlapping and unique OTUs (operational taxonomic units) in largemouth bass (*Micropterus salmoides*) fed the diets containing different supplementation levels of fish protein hydrolysate (FPH).

As shown in Figure 4, the number of operational taxonomic units (OTUs) shared by each group of the samples was 205. The number of unique OTUs in FPH-0, FPH-10, FPH30, and FPH-50 groups were 823, 118, 182, and 58, respectively. Of these, FPH-10 group possessed the largest number of unique OTUs, and FPH-50 group possessed the least number of OTUs. In Table 8, the related alpha diversity index was used to evaluate the effects of FPH on the diversity, richness, and coverage of largemouth bass. The 100% of coverage indicated that almost all microflora phylotypes present in the intestine content samples were notarized. The diversity of the intestinal microflora was estimated by the shannon and simpson indexes, and the richness of the intestinal microflora was estimated by the ace and Chao1. Overall, the Shannon, ace, and Chao1 indexes in the intestine of the FPH-0 treatment were significantly higher than those in FPH-10 and FPH-50 treatments (*p* < 0.05), whereas no significant difference was observed between FPH0 and FPH30 treatments (*p* > 0.05). However, there were no significant differences in Simpson index among all the treatments (*p* > 0.05).

**Table 8:** Alpha diversity analysis of the intestinal microflora of largemouth bass (*Micropterus salmoide*s) fed the diets containing different supplementation levels of fish protein hydrolysate (FPH)^*^.

**Items**	**Diets**			
	**FPH-0**	**FPH-10**	**FPH-30**	**FPH-50**
**Diversity estimators**				
Shannon	2.26 ± 0.17^a^	1.64 ± 0.12^b^	1.97 ± 0.19^ab^	1.55 ± 0.24^b^
Simpson	0.33 ± 0.05	0.24 ± 0.06	0.30 ± 0.02	0.28 ± 0.05
**Richness estimators**				
Ace	415.83 ± 76.04^a^	259.90 ± 23.75^b^	331.23 ± 9.96^ab^	209.26 ± 15.51^b^
Chao1	435.36 ± 66.12^a^	269.64 ± 22.85^b^	327.68 ± 5.70^ab^	237.04 ± 22.58^b^
**Coverage estimators**				
Coverage	1.00 ± 0.00	1.00 ± 0.00	1.00 ± 0.00	1.00 ± 0.00

* *Values are the mean ± SD (n=3) of three replicates. Values within the same row with different superscript small letters are significantly different (P <0.05)*.

#### Analysis of Intestinal Microflora Structure

**Figure 5:**
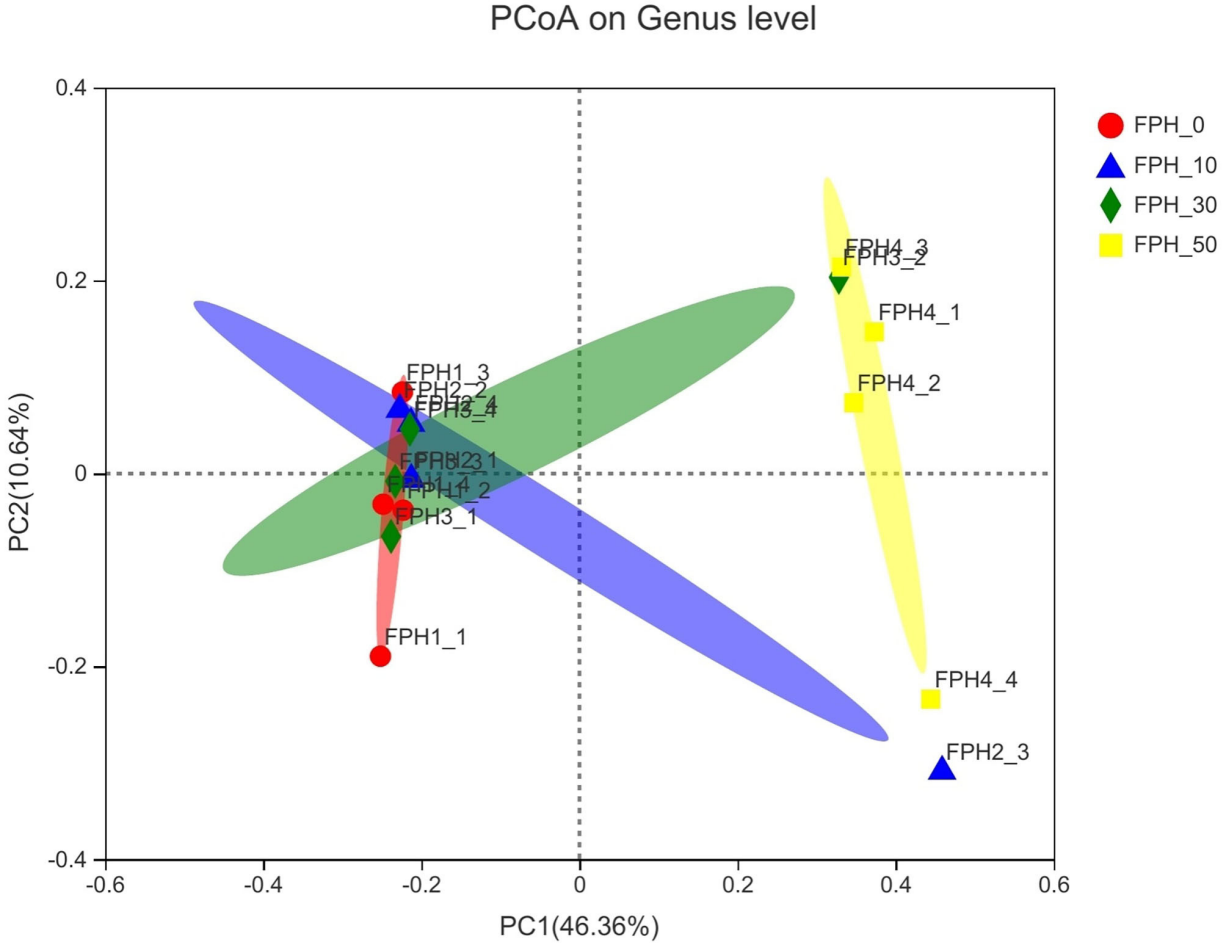
Principal coordinate analysis (PCoA) analysis of the unweighted UniFrac scores of intestinal microflora of largemouth bass (Micropterus salmoides) fed the diets containing different supplementation levels of fish protein hydrolysate (FPH).

**Figure 6:**
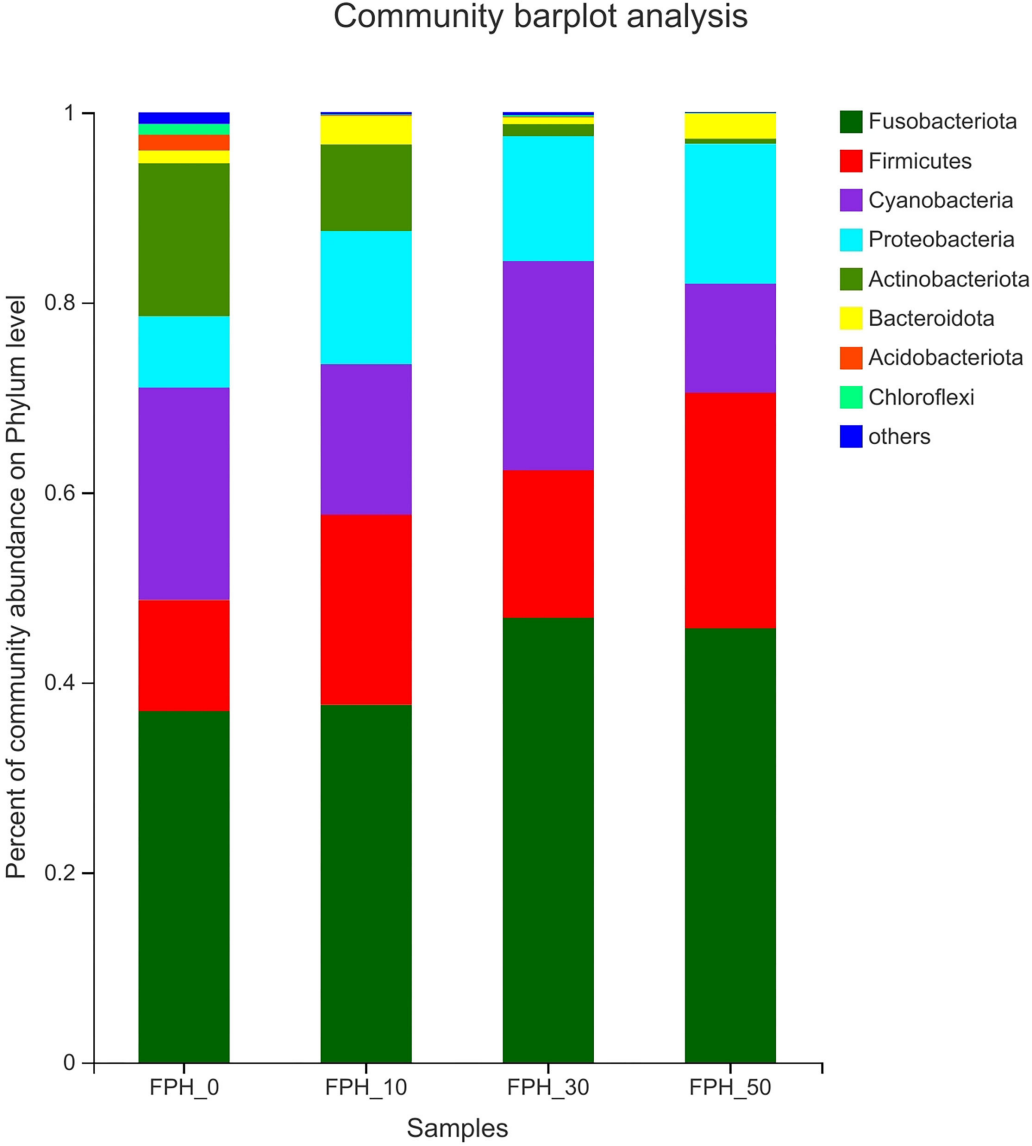
Histogram of relative abundance of intestinal microflora of largemouth bass (*Micropterus salmoides*) fed the diets containing different supplementation levels of fish protein hydrolysate (FPH) at the phylum level.

Principal coordinate analysis (PCoA) revealed that intestinal content samples obtained from the FPH-50 treatment clustered into two regions, and there was no crossover with FPH-0, FPH-10, and FPH-30 treatments (Figure 5). The composition of the top nine intestinal microflora at the phylum level of the relative abundance of juvenile largemouth bass in each treatment is listed in Figure 5. The main intestinal microflora of all groups comprised of *Fusobateriota, Firmicutes, Cyanobacteria*, and *Proteobacteria*. In comparison to the control group (FPH-0), the relative abundance of *Actinobacteriota* decreased significantly in FPH-10, FPH-30, and FPH-50 groups, whereas the variation tendency of the phylum *Proteobacteria* was opposite to that of the *Actinobacteriota* (Figure 6). The highest *Firmicutes* relative abundance and lowest *Bacteroidota* relative abundance was observed in the FPH-30 group, although there was no significant difference with other groups (*p* > 0.05). The ratio of *Firmicutes/Bacteroidota* in the FPH-30 group was significantly higher compared to the other groups (*P* < 0.05) (Table 9).

**Table 9:** Relative abundance of *Firmicutes, Bacteroidetes* and Ratio of *Firmicutes/Bacteroidota* in the intestine of largemouth bass (*Micropterus salmoide*s) fed the diets containing different supplementation levels of fish protein hydrolysate (FPH)^*^.

**Items**	**Diets**			
	**FPH-0**	**FPH-10**	**FPH-30**	**FPH-50**
*Firmicutes*	0.12 ± 0.02	0.19 ± 0.08	0.20 ± 0.05	0.10 ± 0.02
*Bacteroidota*	0.05 ± 0.01	0.05 ± 0.02	0.01 ± 0.00	0.04 ± 0.01
Ratio of *Firmicutes/Bacteroidota*	2.59 ± 0.43^b^	4.70 ± 1.56^b^	21.94 ± 3.24^a^	2.92 ± 1.22^b^

* *Values are the mean ± SD (n = 3) of three replicates. Values within the same row with different superscript small letters are significantly different (P <0.05)*.

**Figure 7:**
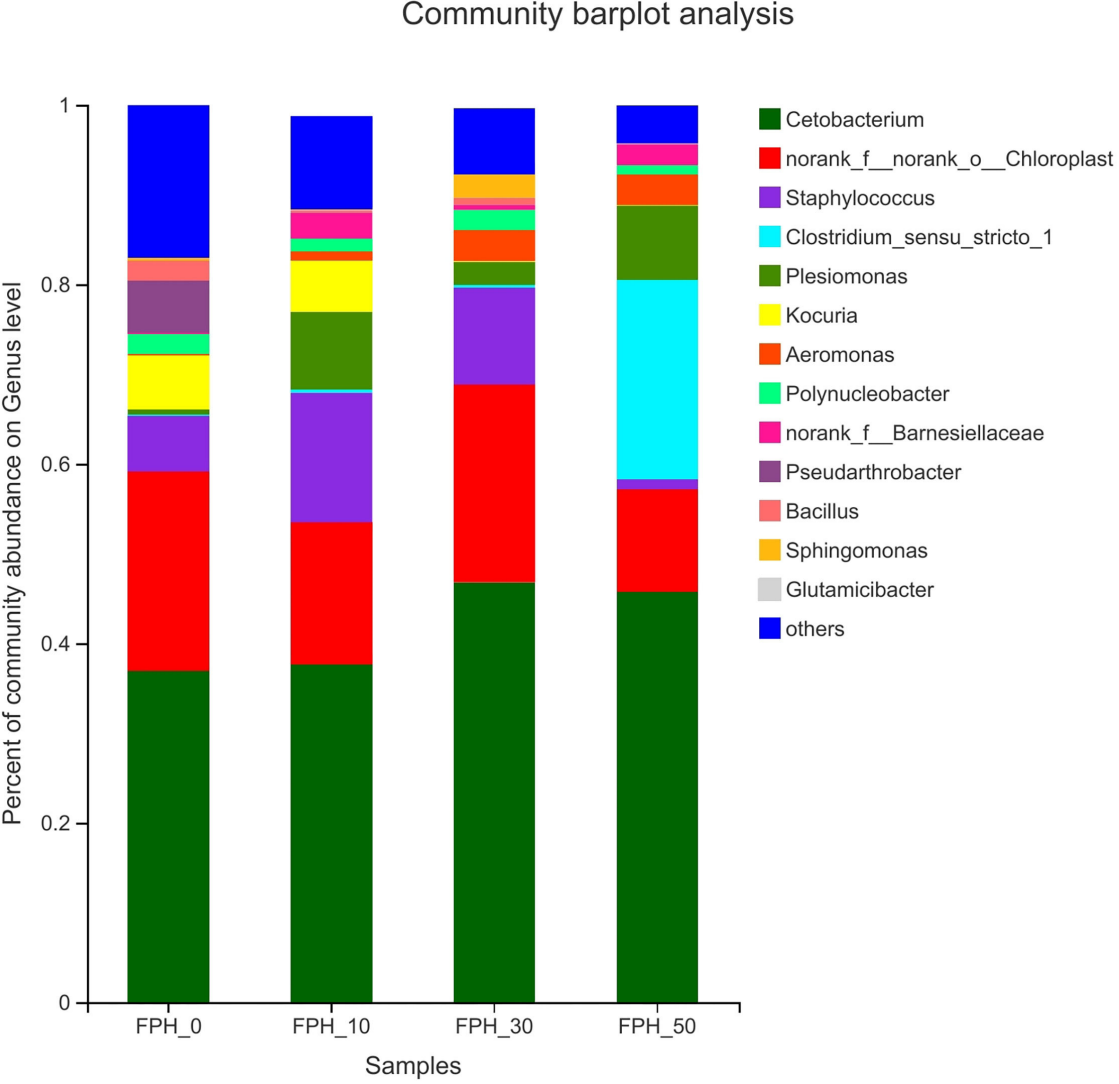
Histogram of relative abundance of intestinal microflora of largemouth bass (*Micropterus salmoides*) fed the diets containing different supplementation levels of fish protein hydrolysate (FPH) at the genus level.

The composition of the top thirteen intestinal microflora at the genus level of the relative abundance of juvenile largemouth bass in each treatment is presented in Figure 7. At the genus level, the dominant microflora in the control group were *Cetobaterium, Staphylococcus, Kocuria*, and *Pseudarthrobacter* based on the relative abundance. *Cetobaterium, Staphylococcus*, and *Plesiomonas* were the dominant microflora in the intestine of juvenile largemouth bass in the FPH-10, FPH-30, and FPH-50 groups dependent on the relative abundance. Compared to the control group, *Pseudarthrobacter* species increased significantly in the FPH-10, FPH-30, and FPH-50 groups. The *Cetobaterium* relative abundance was obviously lifted in the FPH-30 group. The *Kocuria* relative abundance was not detected in the FPH-30 and FPH-50 groups.

## Discussion

In the current trial, further less fish meal (300 g/kg fish meal in the basal feed) were applied in the control (FPH-0) group, and the results indicated that FPH was capable of substituting 5.3–16.3% of dietary fish meal without restraining the growth and feed conversion. Furthermore, 30 g/kg FPH addition, replacing 16.3% of dietary fish meal, made largemouth bass acquire the maximal WGR, SGR, and PER and minimal FCR, suggesting that when viewed at the growth performance and feed utilization, 30 g/kg FPH in largemouth bass feed has realized the fish meal-sparing effects. Inversely, the minimal WGR, SGR, and PER and maximal FCR resulting from 50 g/kg FPH addition, replacing 27.3% of dietary fish meal, has demonstrated that negative fish meal-sparing effects were observed when FPH was unduly added to replace the fish meal for largemouth bass. Also, the variation trend signified that there was a dose-dependent relation between the growth performance and feed utilization and FPH replacing fish meal for largemouth bass, which has been reported for common carp (*Cyprinus carpio*) (40), pike silverside (*Chirostoma estor*) (41), and African catfish (*Clarias gariepinus*) (27). In other researches, no negative growth effects or no dose-dependent relations were reflected in fish fed with the diets containing different levels of the same hydrolysate. In an Asian seabass feed with no fish meal, increasing FPH addition from 10 g/kg to 40 g/kg did not affect the growth performance (42). Analogously, in another study on the application of FPH in Atlantic cod feed conducted by Aksnes et al. (43), when FPH replaced one-third of the fish meal protein, it created a growth difference in the FPH-supplemented groups compared to the FPH-free group. This discrepancy was probably due to the difference from the fish meal contents and type, the peptide size distribution, and the total amount of free amino acids and short chain peptides in the FPH.

Results from our study revealed that marked increment in WGR and feed utilization of fish in the 30 g/kg FPH supplemented group might be partially ascribed to their higher whole-body protein contents because protein is a dominating component of dry-weight basis of fish tissue, thereby determining the growth performance of fish (44). Analogical observations have been seen in Atlantic cod (45), Nile tilapia (46), and turbot (47). Leal et al. (46) found that Nile tilapia fed a diet containing 30 g/kg fish shrimp protein hydrolysate (SPH) had significantly higher protein content compared to fish fed higher levels of protein hydrolysate, while Zheng et al. (47) found that 37 g/kg ultrafiltered fish protein hydrolysate replacing fish meal in the turbot diet had significantly higher protein content compared to fish-meal-based diet. However, on the opposite side, researches on red sea bream and olive flounder reported no effects on the whole-body protein contents (22). Differences from the species, FPH sources, and FPH inclusion levels could have an impact on the effects of dietary FPH on protein deposition. In a deep sense, protein deposition *in vivo* hinges upon protein synthesis ability (48) for aquatic animals, which is reflected in the relative expression of protein synthesis genes. Increasing evidence has shown that hyperactivated TOR signal pathway would promote the rate of protein translation initiation and accelerate the protein translation step of elongation by elevating the expression levels of P13K, AKT, S6K, and 4E-BP1 (49, 50). The observations in the current study suggested that appropriate FPH (30 g/kg) replacing fish meal in largemouth bass feed activated TOR signal pathway through upregulating the expression of S6K and 4E-BP1 in the liver to facilitate the protein synthesis, which was accompanied by increased WGR and SGR. The discrepant regulatory pattern was reported by the previous study in tiger puffer (51), which found that only the 4E-BP1 expression variation trends in muscle among all treatments were in line with elevated WGR and SGR. This may be due to the tissue difference between the two studies. Consequently, the potential mechanisms on FPH supplementation regulating relative expressions of protein deserve further study.

Amino acid response (AAR) signal pathway and GH-IGF axis are the two complementary mechanisms affecting the response status of TOR signal pathway. AAR signal pathway is regarded as the major monitoring indices of individual cellular nutritional perception, mainly including solute carrier family (SLC). Of these, the small peptide transporter (SLC15A1/PepT1, SLC15A2/PepT2) is the functional unit responsible for absorption and transport of small peptides (dipeptide and tripeptide) in the small intestine of animals, and the expression level of SLC15A1/PepT1 in small intestinal epithelial cells will directly reflect the utilization efficiency of proteins in animals (52). The result showed that 30 g/kg FPH upgraduated the expression level of SLC15A1/PepT1 and SLC15A2/PepT2 in the intestine. Similar to SLC15A1/PepT1 and SLC15A2/PepT2, the expressions of AA transporters including SLC7A5, SLC7A8, and SLC38A2 had significantly increased in the intestine of fish fed the 30 g/kg FPH diet, which suggested that the intestine of largemouth bass could have a positive response mechanism on the uptake and absorption of peptides and free AAs through appropriate level of FPH regulating peptide and AA transporters, thereby participating in protein synthesis. The finding was consistent with previous studies in tiger puffer, which showed that FPH supplementation could elevate the positive expressions of SLC1A1 (excitatory amino acid transporters, EAAT3), SLC36A1 (proton-coupled amino acid transporter 1, PAT1), and SLC7A6 (y^+^ L-type amino acid transporter 2, y^+^LAT2) in different membrane surfaces of intestinal epithelial cells to promote the absorption of these free amino acids into the bloodstream or tissues (51). Furthermore, when the FPH addition level (50 g/kg for largemouth bass in this study) was beyond the optimum level, the intestine of largemouth bass could have a negative feedback regulation on the expressions of peptide and AA transporters, thereby affecting the nutrient absorption and growth. In contrast, Wei et al. (19) reported that although the expression levels of SLC15A1/PepT1, SLC6A19 (B^0^ neutral amino acid transporter 1, B^0^AT1), SLC36A1 (proton-coupled amino acid transporter 1, PAT1) in proximal or distal intestine decreased in turbot fed the high level (180 g/kg) of FPH diet, the nutrient absorption and growth were not affected. The discrepancy might be attributed to the hypothesis that the peptide molecular weight distribution of FPH resulted in differential responses of amino acid transporters. The molecular weight <1,000 Da of FPH in our study accounted for 81.1%, whereas the molecular weight <1,000 Da of FPH in the study of Wei et al. (19) accounted for 91.92%, which implied that more small peptide supplied by FPH would arouse the negative feedback effect on the uptake and absorption of peptides for fish. In addition, the GH-IGF axis is considered as the main controller and indicator for systemic nutritional sensing status (53, 54). Emerging evidence from studies has demonstrated that activated GH-IGF signaling could stimulate the TOR pathway through elevating the gene expression level of PI3K/AKT, thereby improving body growth (55, 56). Previous investigations on largemouth bass (57) has verified that amino acid-deficiency diets depressed GH–IGF signaling by decreasing the expression of PI3K/AKT and further suppressed the TOR signaling pathway, which repressed protein anabolism and restrained the growth. Instead, we found that 30 g/kg FPH replacing fish meal in largemouth bass feed elevated the higher GH and IGF-1 expression levels, thereby activating the TOR signaling pathway, which might be an effective feedback mechanism that copes with decreasing fish meal in diets. The above findings also implied that appropriate FPH replacing fish meal in largemouth bass feed could not produce the amino acid deficiency. Taken together, the sparing effect of fish meal could be realized based on FPH accompanied with the ability to promote the protein synthesis.

To obtain the more productive and healthier fish, intestinal health has become the common research target of many researchers, particularly for fish meal substitution research (58–60). There are precise exhibitions which appropriate that FPH addition exhibited an underlying capacity of promoting the nonspecific immune function based on hematological index and hepatic parameters, such as elevated Ig levels (30, 61, 62). Hereof, our study findings supplied the first and preliminary evidence of enhanced immune functions in the intestine of largemouth bass fed with appropriate FPH. As can be seen from the angel of oxidation resistance, the present results identified the enhancement of oxidation resistance by optimal dietary FPH inclusion (30 g/kg), as evidenced by increased activities of SOD, POD, CAT, GSHPx, and T-AOC, reduced MDA contents and downregulated expression levels of SOD and GSHPx, which suggested that FPH could be viewed as a potential antioxidant for fish feed. In terms of antiinflammatory ability, 30 g/kg FPH replacing fish meal in large bass feed could markedly downregulate the expressions of IL-15 and upregulate the expressions of IL-1β and TNFβ of the intestine, which are two critical inflammatory cytokines for largemouth bass (63). This suggests that FPH also modulates the immune response of fish through regulating the expression of cytokines. Moreover, from the cellular point of view, to effectively respond to apoptosis is a sign of immunity enhancement for fish (64). The caspase family is critical in mediating apoptosis as an endogenous pathway. Through the mitochondrial pathway, endogenous apoptosis signals activate the initiating caspases (Caspase8 or Caspase9) and key molecules of downstream signal transduction pathway (Caspase3) to perform apoptosis (65, 66). Our gene function study notarized the markedly repressed expression of Caspase3 and Caspase9 of the intestine by 30 g/kg FPH supplementation, indicating that FPH could inhibit apoptosis and maintain cell homeostasis. Mindfully, the immunoenhancing properties of FPH for the intestine of largemouth bass seem to be dose-dependent, where 50 g/kg FPH supplementation exerts a downregulating effect on intestinal health. Future studies should reveal the role of FPH in affecting intestinal function, as well as the mechanism on regulating the intestinal immunity.

In addition to oxidation resistance, antiinflammatory ability and apoptosis research on intestinal health of fish had gradually begun to focus on intestinal microflora (67, 68). The previous study revealed that *Proteobacteria, Actinobacteria, Firmicute*, and *Cyanobacteria* were the top four dominant bacterial phyla in largemouth bass fed the diets with different starch levels (69). Slightly different with the aforementioned findings, the top four dominant bacterial phylum in this study were presented as *Fusobacteria, Firmicute, Proteobacteria*, and *Cyanobacteria*. This also demonstrated that diet played a crucial key role in the composition of intestinal microbiota (70). Or rather, we provided the first report of altered intestinal microbiota composition in largemouth bass fed with FPH replacing fish meal. Our alpha diversity analysis manifested the reduction of diversity and richness by FPH addition, as evidenced by decreased value of Shannon, Simpson, ace, and Chao1. This change trend was also seen from the microbial community structure at the phylum level. Among the top four dominant bacterial phylum, *Proteobacteria* was more sensitive to FPH, and its abundance presented a marked increase after FPH addition (especially 50 g/kg), which could result in host nutritional and metabolic disorders, thereby motivating the intestinal inflammatory responses (71), in accordance with the research on golden pompano (72). In addition, the negative effect confirmed the reduced *Actinobacteria* abundance, which was able to attenuate the beneficial secondary metabolites and aggravate the pathogenicity of pathogens (73). The *Firmicutes*:*Bacteroidetes* ratio is viewed as a key factor on growth via influencing the nutrient transportation and digestion of the intestine (74, 75). Interestingly, in line with the changing situation of WGR, the peak value of *Firmicutes*:*Bacteroidetes* ratio and the lowest of *Bacteroidetes* abundance were seen in largemouth bass fed 30 g/kg FPH, while the valley value of *Firmicutes*:*Bacteroidetes* ratio and the highest of *Bacteroidetes* abundance were seen in largemouth bass fed 50 g/kg FPH. Given these, it was assumed that compared to *Firmicutes, Bacteroidetes* might be the more pivotal factor on boosting nutritional absorption and elevating weight gain for largemouth bass, which was different from common carp (64) on account of the discrepancy of species and breeding conditions. At the genus level, further analysis revealed that the fish in the three FPH treatments had lower abundances of opportunistic pathogens *Staphylococcus* (76) and *Plesiomonas* (77) than fish in the control treatment, which suggested that FPH could reduce the incidence of intestinal disease for largemouth bass to a certain extent. Allowed for the varieties in intestinal antioxidant capacity, antiinflammatory ability and intestinal microflora mentioned above, we have reason to believe that FPH supplementation could improve the intestinal immune mechanisms of largemouth bass to cope with the immunodeficiency produced by fish meal replacement, although the specific regulatory mechanism is unclear and is required to be investigated in future studies.

## Conclusion

The present work revealed that FPH is a nutritious feed ingredient for juvenile largemouth bass, and can be added to a dietary level of 30 g/kg dry matter replacing fish meal without negative effect on growth and feed utilization. FPH addition was effective in activating the TOR signal pathway *via* the AAR signal pathway and GH-IGF axis, thereby promoting protein synthesis. With overall consideration of the changes in intestinal antioxidant capacity, antiinflammatory ability, and intestinal microflora, FPH supplements could improve the intestinal immune mechanisms of largemouth bass to tackle the immunodeficiency produced by fish meal replacement. Further investigation focusing on the specific mechanism on immunoregulation of FPH is necessary for its application in largemouth bass feed.

## Data Availability Statement

The original contributions presented in the study are publicly available. This data can be found here: National Center for Biotechnology Information (NCBI) BioProject database under accession number PRJNA795284.

## Ethics Statement

The animal study was reviewed and approved by the Committee for the Welfare and Ethics of Laboratory Animals of Heilongjiang River Fisheries Research Institute, CAFS.

## Author Contributions

LW and HLi: conceptualization, investigation, and writing - review & editing. ZF and TL: writing the original draft preparation, formal analysis, and resources. DW: formal analysis and investigation. JL: formal analysis. YZ: formal analysis. ZC: formal analysis. XZ and HLiu: project administration and investigation. All authors contributed to the article and approved the submitted version.

## Funding

This research was supported by the National Key R&D Program of China (2019YFD0900200), China Agriculture Research System of MOF and MARA, Central Public-interest Scientific Institution Basal Research Fund, CAFS (2022XT0402), and Central Public-interest Scientific Institution Basal Research Fund, HRFRI (NO. HSY202111Q).

## Conflict of Interest

ZC and TL are employed by Guangdong Xipu Biotechnology Co., Ltd. HLi is employed by New Hope Liuhe Co., Ltd. The remaining authors declare that the research was conducted in the absence of any commercial or financial relationships that could be construed as a potential conflict of interest. The authors declare that the research was conducted in the absence of any commercial or financial relationships that could be construed as a potential conflict of interest.

## Publisher's Note

All claims expressed in this article are solely those of the authors and do not necessarily represent those of their affiliated organizations, or those of the publisher, the editors and the reviewers. Any product that may be evaluated in this article, or claim that may be made by its manufacturer, is not guaranteed or endorsed by the publisher.
